# Association Between Positive Troponin and Fear of Earthquakes: A Retrospective Study

**DOI:** 10.7759/cureus.45938

**Published:** 2023-09-25

**Authors:** Elias M Nabhan, Pascale Salameh, Samer R Nasr

**Affiliations:** 1 Division of Cardiology, Mount Lebanon University Hospital, Beirut, LBN; 2 Division of Cardiology, University of Balamand, Beirut, LBN; 3 Public Health, Institut National de Santé Publique, Epidémiologie Clinique et Toxicologie (INSPECT-LB), Beirut, LBN; 4 Public Health, University of Nicosia Medical School, Nicosia, CYP

**Keywords:** emergency room, stress, myocardial injury, troponin, fear of earthquake

## Abstract

Introduction

The association between natural disasters and cardiovascular events has been well-established. However, the impact of earthquakes on cardiac health, and the role of fear in this association, remains unclear. This study aims to examine the association between positive troponin levels, indicating cardiac ischemia, and fear of earthquakes among Emergency Room patients at a referral center in Beirut, Lebanon.

Materials and methods

This is a retrospective study conducted on patients who presented to the Emergency Room with suspected cardiac symptoms and were ordered a troponin level after the Kahramanmaras earthquake that occurred on the sixth of February 2023 and affected many neighboring countries, including Lebanon. A control group was taken from the same period in 2022 (January-February) and from the period prior to the earthquake (January up to 6 February 2023). Patients were divided into three groups: the first group (group 1) comprised patients who presented during January and February 2022 (period 1). The second group (group 2) included patients who presented from January up to the sixth of February 2023, when the Kahramanmaras earthquake occurred (period 2). The third group (group 3) consisted of patients who presented after the earthquake until the end of February 2023 (period 3). Patients who consented to participate in the study were sent a questionnaire to assess their Fear of Earthquake Scale (FES), chief complaints, date of presentation to the Emergency Room, past medical history, and other socio-demographic data.

Results

Our study involved 1410 participants, with 782 belonging to group 1, 470 to group 2, and 158 to group 3. The mean age was 62.96 ± 17.87 for the total population and 63.9 ± 18.49 for patients of group 3. The number of positive troponin results was higher during period 3 (62% of participants) in comparison to period 2 and 1 (22.1% and 28.5% of participants respectively) (p<0.001). Positive troponin was significantly more common among patients who are non-smokers (53%, p-value <0.001), with a negative family history of premature cardiac diseases (93.9%, p-value <0.05), previously healthy (46.9%, p-value <0.001) and presenting to the Emergency Room for dyspnea or palpitations (17.3% each, p-value <0.001). In addition, patients who tested positive for troponin had a higher mean FES (27.89 ± 1.23 versus 20.47 ± 6.02) and a higher mean age (71.07 ± 14.33 versus 52.25 ± 18.69) in comparison to those who tested negative for troponin (p-value <0.05).

Conclusion

This study suggests that fear of earthquakes may be associated with cardiac ischemia, as indicated by positive troponin levels. Healthcare providers should be aware of the potential impact of natural disasters on cardiovascular health and take measures to address patients’ fears and concerns.

## Introduction

Lebanon has been struck by several earthquakes in the past, but the recent Kahramanmaras earthquake on February 6th, 2023, has increased fear and anxiety among Lebanese residents, particularly the elderly, who are more vulnerable to psychological disorders such as post-traumatic stress disorder, depression, and anxiety. The earthquake has also caused sleep disorders and nightmares for many Lebanese, adding to their already-existing mental and psychological struggles resulting from political instability, economic crisis, and anxiety disorders in recent years.

Studies have shown that earthquakes can have both immediate and delayed cardiovascular effects, such as increased risk of coronary death and its risk factors [1]. The impact of the Christchurch earthquakes in 2010 and 2011 on cardiovascular disease-related hospital admissions and mortality was also observed, where residents living in areas with the highest level of damage had a higher risk of cardiovascular disease-related hospital admissions, myocardial infarction-related hospital admissions, and cardiovascular disease-related mortality in the first year after the earthquake compared to those living in the least damaged areas [2].

The link between emotional stress and acute cardiac events has also been suggested, where various processes such as stress-induced hemodynamic responses, autonomic dysfunction, neuroendocrine activation, inflammatory responses, and platelet activation can lead to the disruption of coronary plaque, myocardial ischemia, cardiac dysrhythmia, and thrombus formation [3]. A study conducted by Watanabe et al. examined the impact of the Niigata earthquake in Japan on the occurrence of pulmonary embolism [4]. They found that in the four weeks following the earthquake, there were nine cases of pulmonary embolism, compared to one case before the earthquake and two cases in a corresponding period in 2003. 

Furthermore, studies have shown a significant increase in overall mortality rates from acute myocardial infarction in disaster areas after earthquakes [5,6]. In addition, other studies found that the magnitude of a disaster, as well as related stress and population aging, may lead to a temporary increase in the incidence of sudden cardiac and unexpected death [7,8].

However, no studies have examined the association of myocardial ischemia represented by the troponin level and fear of earthquakes in Lebanon. Thus, the aim of this study is to investigate the association between positive troponin levels, indicating cardiac ischemia, and fear of earthquakes among Emergency Room patients at a referral center in Beirut, Lebanon. 

## Materials and methods

Study design

A retrospective cross-sectional study was conducted in January and February 2023, during which two earthquakes occurred on February 6 and 20. A control group was taken from the same time period in 2022. The study was conducted on Emergency Room patients at a single referral center in Beirut. Participants who presented after the Kahramanmaras earthquake occurred (February 6, 2023) were given a questionnaire in English and Arabic, either through social media or during follow-up visits in the outpatient department, to assess their level of fear related to earthquakes using the Fear of Earthquake Scale (FES) and to gather information on their chief complaints, date of presentation to the Emergency Room, past medical history, and other socio-demographic data. Only patients who had a cardiac troponin I (cTn I) test ordered and consented to participate were included in the study. Concerning cardiac troponin I (cTn I), values equal to or above 0.04 ng/mL were considered as positive. 

The FES, developed by Prizmić-Larsen et al., was used to assess the level of fear of earthquakes [9]. The scale consists of seven items, and participants rated each item on a 5-point Likert scale, with 1 representing "strongly disagree" and 5 representing "strongly agree". The minimum score possible for each question is 1, and the maximum is 5. The total score is calculated by adding up the scores for each item, ranging from 7 to 35. According to the study done by Prizmić-Larsen et al., the FES had a Cronbach’s alpha of 0.9, in our study, it was 0.87. A higher score indicates a greater level of fear related to earthquakes [9].

Eligibility

The study only included patients who provided their consent and excluded those with an impaired glomerular filtration rate or an active infectious process.

Statistical plan

The data collected were entered into an Excel sheet and analyzed using IBM SPSS Statistics for Windows, Version 22 (Released 2013; IBM Corp., Armonk, New York, United States). Patients were divided into three groups based on the time of presentation to the Emergency Room. The first group (group 1) comprised patients who presented during January and February 2022 (period 1). The second group (group 2) included patients who presented from January to the sixth of February 2023, when the Kahramanmaras earthquake occurred (period 2). The third group (group 3) consisted of patients who presented after the earthquake until the end of February 2023 (period 3).

Descriptive statistics were reported using means and standard deviations (SD) for continuous variables and frequency with percentages for categorical variables.

A statistical bivariate analysis was performed. Student's t-test was used for the continuous variables to compare their means. The Pearson Chi-square (χ2) test / Fisher exact test was used for categorical variables, as appropriate. A p-value less than 0.05 was considered statistically significant.

## Results

Our study involved 1410 participants, with 782 belonging to group 1, 470 to group 2, and 158 to group 3.

The mean age was 62.96 ± 17.87 for the total population and 63.9 ± 18.49 for patients of group 3. Descriptive statistics among the total population are shown in Table 1. The majority of our total population were males (58.8%), belonging to group 1 (55.4%) and testing negative for troponin (69.8%). 

**Table 1:** Descriptive statistics among the total population

Variable	Frequency	Percentage
Gender		
Males	830	58.8
Females	580	41.1
Group		
Group 1	782	55.4
Group 2	470	33.3
Group 3	158	11.2
Troponin		
Positive	425	30.1
Negative	985	69.8

Table 2 demonstrates that the number of positive troponin results was higher during period 3 (62% of period 3 participants compared to 22.1% and 28.5% of period 2 and 1 participants, respectively) suggesting a possible effect of the earthquake on the positivity of the troponin test among patients presenting to the Emergency Room (p<0.001). 

**Table 2:** Variation of troponin results according to the groups

Variable	Negative troponin		Positive troponin		Total		P value
	Frequency	Percentage	Frequency	Percentage	Frequency	Percentage	
Group (period of time)							<0.001
Group 1	559	71.5	223	28.5	782	100	
Group 2	366	77.9	104	22.1	470	100	
Group 3	60	38	98	62	158	100	

The average FES among patients of group 3 was 25.06 ± 5.25. Descriptive statistics of the seven components of the FES are represented in Table 3. 

**Table 3:** Fear of Earthquake Scale (FES) components SD: Standard deviation; FES: Fear of Earthquake Scale

Variable	Mean ± SD
1. I am most afraid of earthquakes.	4.32 ± 1.26
2. It makes me uncomfortable to think about earthquakes.	4.28 ± 1.21
3. My hands become clammy when I think about earthquakes.	2.3 ± 0.50
4. I am afraid of losing my life because of earthquakes.	4.26 ± 1.16
5. When I watch news and stories about earthquakes on social media, I become nervous or anxious.	3.73 ± 0.92
6. I cannot sleep because I’m worrying about earthquakes.	3.11 ± 0.71
7. My heart races or palpitates when I think about earthquakes.	3.06 ± 0.77
Total FES average score	25.06 ± 5.25

Table 4 reveals that in group 3, the majority of patients were males (58%), non-smokers (43%), with no family history of premature cardiac disease (96.2%), previously healthy (57.6%), and those presenting to the Emergency Room for dyspnea (21.5%) followed by palpitations (18.4%) and having a positive troponin (62%). A chi-squared analysis was conducted to study the difference between troponin results and each of these characteristics. There were no significant differences in gender (p-value >0.05); however, a positive troponin was significantly more common among patients who are non-smokers (53%, p-value <0.001), with a negative family history of premature cardiac diseases (93.9%, p-value <0.05), previously healthy (46.9%, p-value <0.001) and those presenting to the Emergency Room for dyspnea or palpitations (17.3% each, p-value <0.001).

**Table 4:** Characteristics of the group 3 participants according to troponin results p-value < 0.05 was considered statistically significant.

Variable	Positive troponin (n=98, 62%)		Negative troponin (n=60, 38%)		Total (n=158)		p-value
	Frequency	Percentage	Frequency	Percentage	Frequency	Percentage	
Gender							0.31
Males	59	60.2	33	55	92	58.2	
Females	39	39.8	27	45	66	41.8	
Total	98	100	60	100	158	100	
Smoking Status							<0.001
Non-smoker	52	53.1	16	26.7	68	43	
Daily smoker	18	18.4	40	66.7	58	36.7	
Occasional smoker	27	27.6	4	6.7	31	19.6	
Ex-smoker	1	1	0	0	1	0.6	
Total	98	100	60	100	158	100	
Family history of premature cardiac diseases							<0.05
Yes	6	6.1	0	0	6	3.8	
No	92	93.9	60	100	152	96.2	
Total	98	100	60	100	158	100	
Past medical history							<0.001
Previously healthy	46	46.9	45	75	91	57.6	
Hypertension	26	26.5	12	20	38	24.1	
Diabetes mellitus	10	10.2	0	0	10	6.3	
Dyslipidemia and diabetes mellitus	7	7.1	1	1.7	8	5.1	
Coronary artery disease	5	5.1	0	0	5	3.2	
Dyslipidemia	3	3.1	2	3.3	5	3.2	
Dyslipidemia and arrhythmias	1	1	0	0	1	0.6	
Total	98	100	60	100	158	100	
Chief complaint upon presentation to the Emergency Room							<0.001
Dyspnea	17	17.3	17	28.3	34	21.5	
Palpitations	17	17.3	12	20	29	18.4	
Epigastric pain	8	8.2	19	31.7	27	17.1	
Chest pain	24	24.5	2	3.3	26	16.5	
Dizziness	20	20.4	0	0	20	12.7	
High blood pressure	10	10.2	10	16.7	20	12.7	
Diaphoresis	2	2	0	0	2	1.3	
Total	98	100	60	100	158	100	

**Figure 1:**
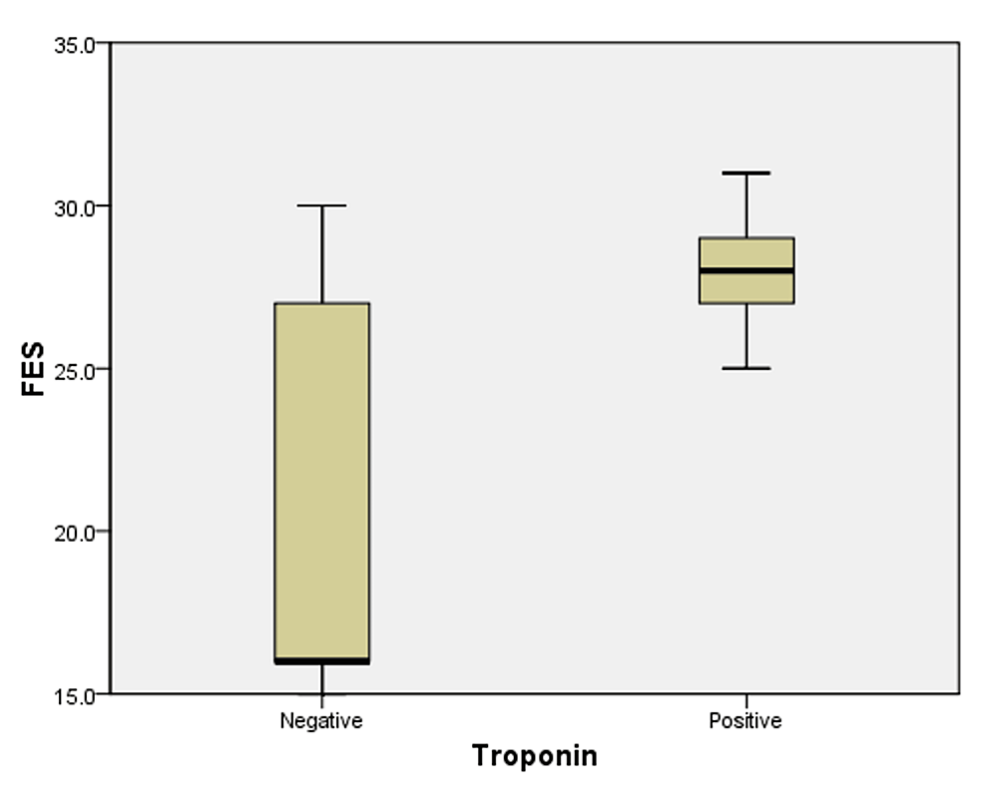
Variation of Fear of Earthquake Scale (FES) according to troponin results among patients of group 3 A t-test was conducted to determine statistical significance. There was a statistically significant variation of the FES according to troponin results among patients of group 3 (p<0.001). FES: Fear of Earthquake Scale.

**Figure 2:**
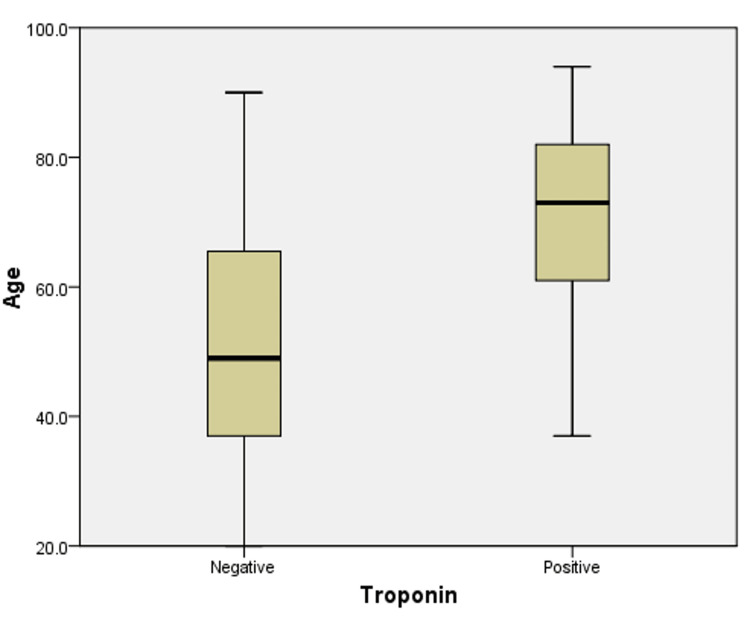
Variation of age according to troponin results among patients of group 3 A t-test was conducted to determine statistical significance. There was a statistically significant variation of age according to troponin results among patients of group 3 (p<0.001).

As shown in Figure 1 and Table 5, among patients of group 3, the FES ranged between 15 and 31. In addition, the FES ranged between 25 and 31, with a mean of 27.89 ± 1.23 among those who tested positive for troponin, and between 15 and 30, with a mean of 20.47 ± 6.02 among those who tested negative for troponin. This indicates a higher FES among patients of group 3 with positive troponin in comparison to those with negative troponin (p-value <0.001) suggesting that fear of earthquakes may be associated with myocardial injury represented by positive troponin. On the other hand, Figure 2 and Table 5 indicate that the age of patients in group 3 ranged between 37 and 94 years old with a mean of 71.07 ± 14.33 among those who tested positive for troponin and between 20 and 90, with a mean of 52.25 ± 18.69 among those who tested negative for troponin. This indicates a greater mean of age among patients who tested positive for troponin in comparison to those who tested negative. 

**Table 5:** Troponin results according to Fear of Earthquake Scale (FES) and age of group 3 patients A t-test was conducted to determine the statistical significance. p-value < 0.05 was considered as statistically significant. SD: Standard deviation; FES: Fear of Earthquake Scale

Variable	Positive troponin (n=98)	Negative troponin (n=60)	p-value
	Mean ± SD	Mean ± SD	
Age	71.07 ± 14.33	52.25 ± 18.69	0.007
FES	27.89 ± 1.23	20.47 ± 6.02	<0.001

## Discussion

This study examined the association between myocardial injury, as reflected by troponin levels, and fear of earthquakes by separating the population based on the date of presentation to the Emergency Room into groups before and after the Kahramanmaras earthquake occurred at a referral center in Beirut, Lebanon.

Our findings suggest that the period after the earthquake occurred (period 3) was associated with a higher incidence of positive troponin levels, as 62 % of patients who presented to the Emergency Room during this period had a positive troponin, while only 22.1% and 28.5% tested positive for troponin during period 1 and period 2 (p-value <0.001). This result is in line with a study conducted by Tsai et al. which examined the impact of stress resulting from the Ji-Ji, Taiwan earthquake that occurred on September 21, 1999, at 1:47 a.m., on acute myocardial infarction in six counties near the epicenter [10]. Hospitalization rates for acute myocardial infarction increased during the six weeks following the earthquake, with a significantly higher number of patients hospitalized during that period compared to the same duration in the previous year. In a similar fashion, Watanabe et al. conducted a study in Japan, which examined the impact of the Niigata earthquake on the occurrence of pulmonary embolism and found that in the four weeks following the earthquake, there were nine cases of pulmonary embolism, compared to one case before the earthquake and 2 cases in a corresponding period in 2003 [4].

During period 3, positive troponin was significantly more common among patients who are non-smokers (53%, p-value <0.001), with a negative family history of premature cardiac diseases (93.9%, p-value <0.05), previously healthy (46.9%, p-value <0.001), presenting to the Emergency Room for dyspnea or palpitations (17.3% each, p-value <0.001).

In addition, patients who tested positive for troponin during period 3 had a higher mean FES (27.89 ± 1.23 versus 20.47 ± 6.02) and mean age (71.07 ± 14.33 versus 52.25 ± 18.69) in comparison to those who tested negative for troponin (p-value <0.05). This suggests a possible association between the fear of earthquakes and myocardial injury represented by positive troponin, especially among the elderly. However, further studies are necessary to assess the “dose-effect” relationship between fear and myocardial injuries.

The observed difference in troponin results before and after the earthquake may be attributed to the significant stress experienced by individuals living in areas that were affected by the 7.8 Magnitude Kahramanmaras earthquake such as Lebanon. This particular earthquake occurred on February 6, 2023, at 3:20 am, catching many people off guard while they were sleeping, and in a region where earthquakes are infrequent. Moreover, the fear and anxiety of affected citizens were further intensified by another strong 6.3 Magnitude earthquake that struck southern Turkey 15 days later and was felt in Lebanon [11].

This is consistent with a study conducted by Brown on disparate effects of the 1989 Loma Prieta and 1994 Northridge earthquakes on hospital admissions for acute myocardial infarction which found a significant increase in admission rates for acute myocardial infarction in Los Angeles on the day of the Northridge earthquake in 1994, compared to the average seven days before the earthquake [12]. The study also reported a greater than 80% increase in the risk of acute myocardial infarction admission on the day of the earthquake, compared to the same date in 1995. These results suggest that extreme emotional stress, in addition to the stress of awakening, may lead to an enhanced triggering of acute myocardial infarction.

In their review titled "Natural disaster plus wake-up time: A deadly combination of triggers," Kloner et al. further noted that it is not uncommon for cardiac events to increase when a large population is suddenly awakened by a life-threatening event [13]. The combination of a natural disaster and the wake-up time can be particularly dangerous. The increase in cardiac events following the Northridge earthquake was attributed to the sudden awakening to a frightening event, which led to a massive surge of sympathetic and catecholamine activity. Similarly, Yousuf et al. emphasized that all natural disasters contribute to cardiovascular stress, and while natural disasters are unavoidable, disaster preparedness can significantly reduce their devastating impact on human life [14].

This study is the first of its kind in Lebanon to explore this relationship, and it adds to the growing body of literature on the cardiovascular effects of stress which has gained a special significance in the wake of recent events in Lebanon. In fact, the past three years have witnessed numerous stressful events in Lebanon, including the devastating explosion at Beirut’s port on August 4, 2020, which claimed over 219 lives and caused widespread destruction. Additionally, the country is currently grappling with a severe socio-economic crisis that the World Bank has labeled a "deliberate depression," ranking it among the most significant financial crises since the mid-nineteenth century, leading to the Lebanese currency depreciation by more than 95% and unavailability of money deposits from banks [15].

Thus, policymakers involved in designing cardiovascular disease prevention strategies should prioritize continuous monitoring and implement preventive measures such as building codes, medical readiness, public education, and mental health programs to mitigate the impact of such disasters on cardiovascular health. Based on our findings, it is also important to further investigate this relationship in other countries that have experienced earthquake-related trauma, particularly Syria and Turkey where further studies in these regions could provide valuable insights into the impact of fear of earthquakes on cardiovascular health.

Our study had several limitations. Firstly, it was conducted at a single center in Beirut, Lebanon, and may not be representative of the entire population of the country. Secondly, the sample size of the study was relatively small, which may limit the statistical power and the ability to draw robust conclusions. Thirdly, the study only measured troponin levels as a marker of myocardial injury and did not assess other markers or imaging modalities that could provide a more comprehensive assessment of cardiac function. Lastly, the FES has not yet been validated in Lebanon and may not accurately capture the level of fear experienced by patients.

## Conclusions

In conclusion, this study demonstrated that patients who presented after the Kahramanmaras earthquake occurred and who tested positive for troponin had a significantly higher mean score on the FES compared to those who tested negative. This suggests that fear of earthquakes may be associated with myocardial injury represented by a positive troponin level in comparison to the period before the earthquake occurred especially among the elderly. However, more studies are necessary to confirm this finding, and measures should be taken by authorities to mitigate the effect of this stressful factor on the general population.
